# Application of Partial Internal Transcribed Spacer Sequences for the Discrimination of* Artemisia capillaris* from Other* Artemisia* Species

**DOI:** 10.1155/2016/7043436

**Published:** 2016-05-30

**Authors:** Eui Jeong Doh, Seung-Ho Paek, Guemsan Lee, Mi-Young Lee, Seung-Eun Oh

**Affiliations:** ^1^Division of Biological Sciences, Konkuk University, Seoul 143-701, Republic of Korea; ^2^Department of Herbology, Wonkwang University, Iksan 570-749, Republic of Korea; ^3^Korea Institute of Oriental Medicine, Daejeon 305-811, Republic of Korea

## Abstract

Several* Artemisia* species are used as herbal medicines including the dried aerial parts of* Artemisia capillaris*, which are used as Artemisiae Capillaris Herba (known as “Injinho” in Korean medicinal terminology and “Yin Chen Hao” in Chinese). In this study, we developed tools for distinguishing between* A. capillaris* and 11 other* Artemisia* species that grow and/or are cultured in China, Japan, and Korea. Based on partial nucleotide sequences in the internal transcribed spacer (ITS) that differ between the species, we designed primers to amplify a DNA marker for* A. capillaris*. In addition, to detect other* Artemisia* species that are contaminants of* A. capillaris*, we designed primers to amplify DNA markers of* A. japonica*,* A. annua*,* A. apiacea*, and* A. anomala*. Moreover, based on random amplified polymorphic DNA analysis, we confirmed that primers developed in a previous study could be used to identify* Artemisia* species that are sources of Artemisiae Argyi Folium and Artemisiae Iwayomogii Herba. By using these primers, we found that multiplex polymerase chain reaction (PCR) was a reliable tool to distinguish between* A. capillaris* and other* Artemisia* species and to identify other* Artemisia* species as contaminants of* A. capillaris* in a single PCR.

## 1. Introduction

The genus* Artemisia* belongs to the Asteraceae family and is composed of 500 species that are mainly found in Asia, Europe, and North America [1, 2]. Over 350 species in the genus* Artemisia* are grown in Asia, including China, Korea, and Japan [1]. Several* Artemisia* species have long been used for the treatment of disease in modern and traditional medicine [2, 3]. For example, the dried aerial parts of* A. capillaris* are used as Artemisiae Capillaris Herba (“Injinho” in Korean medicinal terminology and “Yin Chen Hao” in Chinese) [4], which controls fever [2], protects the liver [5], and inhibits inflammatory responses [6]. However, the dried leaves of* A. capillaris* are often mistaken for those of* A. japonica.* Moreover, young* A. capillaris* leaves that are harvested in early spring are similar to those of* A. argyi* and* A. princeps* [7], which are sources of Artemisiae Argyi Folium (“Aeyup” in Korean and “Ai Ye” in Chinese) that is used for the treatment of pain, vomiting, and bleeding in the uterus [8].

Because of the morphological similarities among the dried and/or sliced shoots and leaves of* Artemisia* species, some are traded as other species in traditional herbal medicine markets [5, 7]. To resolve this problem, various molecular biology techniques that are based on plant genetic information, such as gene nucleotide sequences (*rbcL*,* matK*, or a combination of both), have been used for plant identification and authentication, including medicinal plants [3]. Other gene sequences have been used to discriminate specific medicinal plants from an adulterant or substitute, for example, the* trnL-F* intergenic spacer for* Coptis* spp.,* matK* for* Rheum* spp., and* psbA*-*trnH* for* Phyllanthus* spp. [9–11]. Internal transcribed spacer (ITS) sequences are effective discriminatory tools, and the ITS2 region in particular can be used as a universal DNA barcode for identifying plants and animals [12], including medicinal plants in the family Fabaceae [13] and genus* Artemisia* [14].

Random amplified polymorphic DNA- (RAPD-) based DNA markers have been used previously for authenticating medicinal plants [3]. In a previous study conducted on six* Artemisia* species that mainly grow and/or are cultured in Korea (*A. princeps*,* A. argyi*,* A. capillaris*,* A. iwayomogi*,* A. japonica*, and* A. keiskeana*), we discriminated both* A. princeps* and* A. argyi* from other* Artemisia* species using a sequence-characterized amplified region (SCAR) marker, which was based on RAPD results [7]. Using the same method, we identified* A. iwayomogi*, which is a source of Artemisiae Iwayomogii Herba (“Haninjin” in Korean medicinal terminology) that has been prescribed as a substitute for Artemisiae Capillaris Herba in Korea [15]. However, we were unable to discriminate* A. capillaris* from* A. japonica* using the RAPD-based method [5, 7].

In this study, we discriminated* A. capillaris* from other* Artemisia* species, particularly* A. japonica*, by exploiting sequence differences in a specific region of the ITS. We used 12* Artemisia* species, six from our previous study and 6 additional* Artemisia* species, which grow and/or are cultivated in China, Korea, and Japan:* A. asiatica*,* A. montana*, and* A. lavandulaefolia*, which are sources of Artemisiae Argyi Folium in China and Korea [4, 16, 17];* A. annua* and* A. apiacea*, which are sources of Artemisiae Annuae Herba (“Chung-ho” in Korean medical terminology and “Qing Hao” in Chinese) [4, 18] used for the treatment of malaria [19]; and* A. anomala*, which is a source of Artemisiae Anomalae Herba (“Yugino” in Korean medicinal terminology and “Liu ji nu” in Chinese) [4, 17] used for the treatment of fever and inflammation [20].* A. anomala* was included to increase the reliability of the discrimination of* A. capillaris*. In addition, we tested the effectiveness of multiplex polymerase chain reaction (PCR) to detect contamination of* A. capillaris* products with those from other* Artemisia* species. We used primers based on ITS sequences to discriminate among the* Artemisia* species and two RAPD-based primer sets to discriminate between* Artemisia* species that are sources of Artemisiae Argyi Folium and Artemisiae Iwayomogii Herba [5, 7].

## 2. Materials and Methods

### 2.1. Plant Materials

The fleshy aerial parts, including the leaves, of* Artemisia* species that grow and/or are cultivated in China, Korea, and Japan were collected (Table 1). The samples were dried at room temperature, frozen, and stored at −80°C. The authenticity of the samples was verified by the Korea Institute of Oriental Medicine (KIOM) and the Department of Herbology, Wonkwang University. The voucher samples were deposited in the KIOM and the Department of Herbology.

### 2.2. Preparation of Genomic DNA

Genomic DNA from each sample was extracted in accordance with the instruction manual for the NucleoSpin® Plant II (Macherey-Nagel, Duren, Germany). To improve DNA quality, phenolic compounds and polysaccharides were removed using 10% cetyltrimethylammonium bromide and 0.7 M NaCl. After the purity and amount of the prepared genomic DNA were determined using a NanoDrop*™* DN-1000 spectrophotometer (Thermo Scientific, Wilmington, DE, USA), the DNA was diluted to 10 ng/*μ*L and stored.

### 2.3. PCR Amplification

#### 2.3.1. Amplification of ITS

A PCR for the amplification of the ITS, including the 5.8S rRNA coding region, was conducted using a T-personal cycler (Biometra, Goettingen, Germany) according to the protocol by White et al. [21]. In brief, 1.2 pmol of ITS1 (5′-TCCGTAGGTGAACCTGCGG-3′) and ITS4 (5′-TCCTCCGCTTATTGATATGC-3′) primers, 1 U* Taq* polymerase (ABgene, Epsom, UK), and 20 ng of genomic DNA extracted from each sample were used for the PCR amplification. During the 35-cycle PCR process, predenaturation was conducted for 5 min at 95°C and denaturation for 30 s at 95°C. The annealing process was conducted for 30 s at 52°C and the extension process for 1 min at 72°C. A final reaction step was conducted for 7 min at 72°C. The amplified products were separated on 1.2% agarose gel and revealed by staining with ethidium bromide (Sigma-Aldrich, St. Louis, MO, USA). The amplified PCR products were analyzed using MyImage (Seoulin Biotechnology, Seoul, Korea) and purified using a LaboPass*™* Gel Kit (Cosmo Genetech, Seoul, Korea).

#### 2.3.2. Amplification of DNA and SCAR Markers

In brief, 1.2 pmol of primers, 1 U* Taq* polymerase (ABgene), and 50 ng of genomic DNA extracted from each* Artemisia* species were used for the PCR amplification. During the 23-cycle PCR process, predenaturation was conducted for 5 min at 95°C and denaturation for 30 s at 95°C. In general, the annealing process was conducted for 30 s at 53.5°C for the amplification of the DNA markers. However, to amplify the DNA markers for* A. capillaris*,* A. japonica*,* A. apiacea*,* A. annua*, and* A. anomala*, this process was conducted for 15–30 s at 54–58°C. The extension process was conducted for 20 s (except for* A. apiacea*, which had 30 s) at 72°C, and a final reaction step was conducted for 5 min at 72°C. To amplify an internal standard for the evaluation of the PCR conduct, a primer set (AYF/AYR) was used to amplify a 94 bp sequence. The amplified products were separated on 1.2% agarose gel and revealed by staining with ethidium bromide (Sigma-Aldrich). The amplified PCR products were then analyzed using MyImage (Seoulin Biotechnology).

#### 2.3.3. Multiplex PCR

For the multiplex PCR amplification, 0.07 pmol of the primers Fb and R7; 0.14 pmol of the primers AYF and AYR; 0.7 pmol of the primer Aam F3; 1.7 pmol of the primers AC F4, ACJ R3, and Aap R2; 3.4 pmol of the primers 2F1, 2F3, AJ F1, AC R3, Aap F1, AA F3, and Aa R4; 1x PrimeSTAR® Max DNA Polymerase (Takara Bio Inc., Kusatsu, Japan); and 20 ng of genomic DNA extracted from each* Artemisia* species were used. During the 30-cycle PCR process, predenaturation was conducted for 10 min at 95°C and denaturation for 10 s at 95°C. The annealing process was conducted for 5 s at 56.5°C and the extension process for 10 s at 72°C. A final reaction step was conducted for 7 min at 72°C. The amplified products were separated on 2% agarose gel and revealed by staining with ethidium bromide (Sigma-Aldrich). In order to amplify an internal standard for the evaluation of the PCR, the AYF/AYR primer set was used to amplify a 94 bp sequence. The amplified PCR products were then analyzed using MyImage (Seoulin Biotechnology).

### 2.4. Nucleotide Sequencing of the PCR Products

The nucleotide sequences of the PCR products were directly determined using the primers ITS1 and ITS4 by Macrogen (Seoul, Korea). In other cases, the PCR products resolved by agarose electrophoresis were cloned using a pGEM®-T Easy Vector System I (Promega, Madison, WI, USA). The nucleotide sequences of the subcloned PCR products were determined by Macrogen.

### 2.5. Alignment of the DNA Sequences and Construction of a Dendrogram

The DNA sequences were manually edited and aligned by ClustalW multiple sequence alignment in BioEdit v7.0.9 (http://www.mbio.ncsu.edu/BioEdit/bioedit.html). A dendrogram was constructed using the neighbor-joining method [22] in the MEGA6 program [23] with 1000 bootstrap iterations. Evolutionary distances were computed using the maximum composite likelihood method [24] in MEGA6.

## 3. Results

### 3.1. Determination and Analysis of ITS Sequences

**Figure 1:**
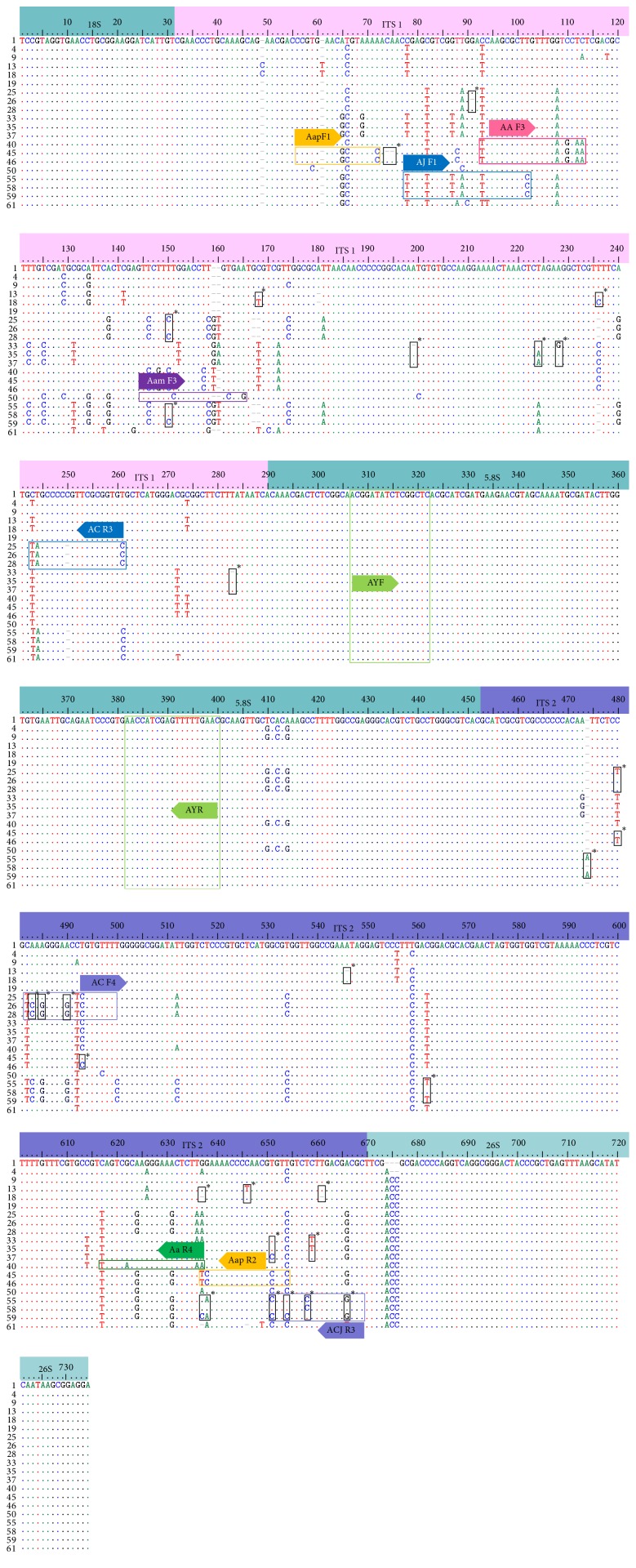
Multiple alignments of nucleotide sequences of the internal transcribed spacer (ITS) among* Artemisia* species. The dots indicate consensus nucleotides and the dashes represent gaps. Numbers represent sample numbers (see Table 1). Bold arrows indicate the primers used to amplify DNA markers of the* Artemisia* species, and colored boxes represent nucleotide sequences as well as the positions of the ITS in the primers. Black boxes with an asterisk indicate variations in the nucleotides within species.

The 726–731 bp nucleotide sequences of the ITS, including the 5.8S region, were determined in 65 samples of 12* Artemisia* species (Table 1). Parts of the ITS sequences of each* Artemisia* species are presented in Figure 1 and were deposited in GenBank (accession numbers KT965653–KT965672). As shown in Figure 1, in the intraspecific samples of five* Artemisia* species (*A. argyi*,* A. capillaris*,* A. iwayomogi*,* A. apiacea*, and* A. japonica*), 4–9 bp differences in the ITS1 and ITS2 sequences were detected. In the case of* A. japonica* (sample numbers 55, 58, and 59), there were 8 bp differences in the ITS2 region and a 1 bp difference in the ITS1 region. These differences resulted mainly from substitutions (mostly base transitions) and a deletion. In* A. apiacea* (sample numbers 45 and 46), two base deletions in ITS1 and two substitutions in ITS2 were detected in sample number 45.

**Figure 2:**
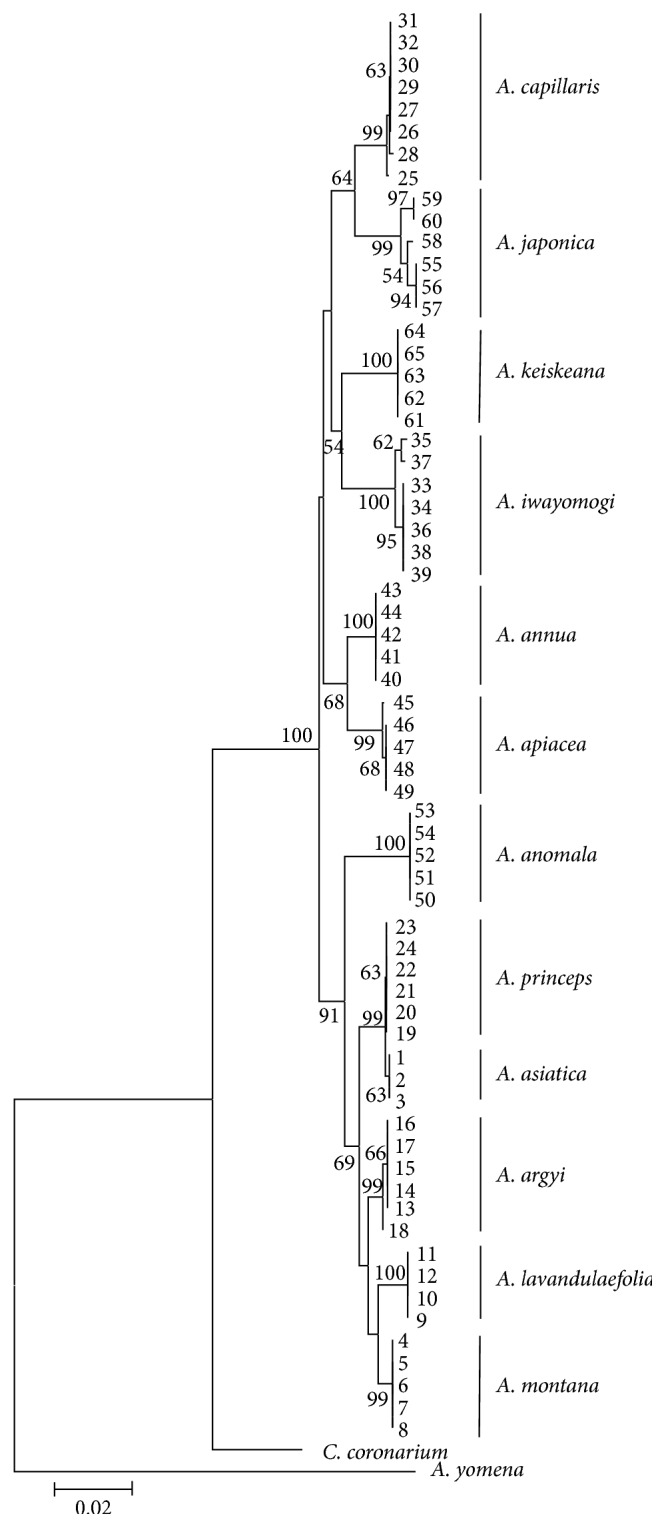
Dendrogram based on the internal transcribed spacer (ITS) sequences presented in Figure 1. ITS sequences of* Aster yomena* (accession number HQ154048.1) and* Chrysanthemum coronarium* (accession number EF577292.1) in GenBank were used as outgroups. The unit of evolutionary distance was the number of base substitutions per site; bootstrap values of over 50% are indicated on the branches of the dendrogram.

To determine whether each* Artemisia* species could be identified by interspecific ITS sequence differences, we constructed a dendrogram based on the ITS sequences. As outgroups, we used GenBank sequences of* Aster yomena* (accession number HQ154048.1) and* Chrysanthemum coronarium* (accession number EF577292.1) in the family Asteraceae, in which* Artemisia* is included (Figure 2). As shown in Figure 2, each* Artemisia* species was classified into a separate group on the dendrogram. All of the* A. japonica* samples that exhibited excessive intraspecific ITS sequence variation were sorted into a group. Fortunately, the* A. capillaris* samples were separate from the* A. japonica* samples on the dendrogram. In addition, both* A. annua* and* A. apiacea*, which are sources of Artemisia Annuae Herba, were classified into the same cluster on the dendrogram (Figure 2). Interestingly,* A. argyi*,* A. princeps*,* A. montana*,* A. lavandulaefolia*, and* A. asiatica*, which are sources of Artemisiae Argyi Folium, were classified into only one cluster.

### 3.2. Discrimination of* A. capillaris* from Other* Artemisia* Species by Differences in ITS Sequences

**Figure 3:**
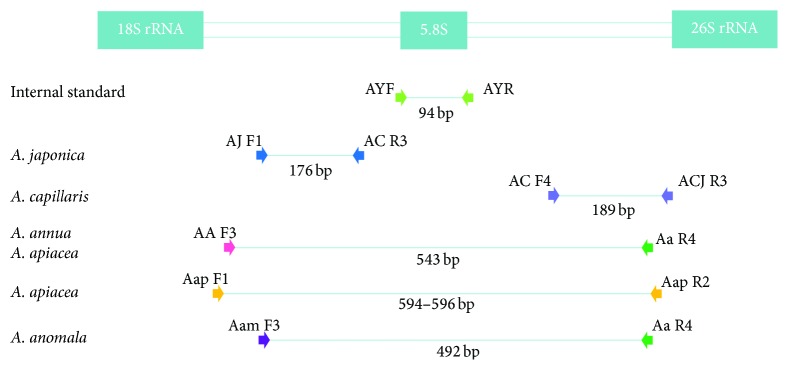
Relative positions of the primers designed to amplify DNA markers of* Artemisia* species on the internal transcribed spacer and the expected size of the polymerase chain reaction products.

**Figure 4:**
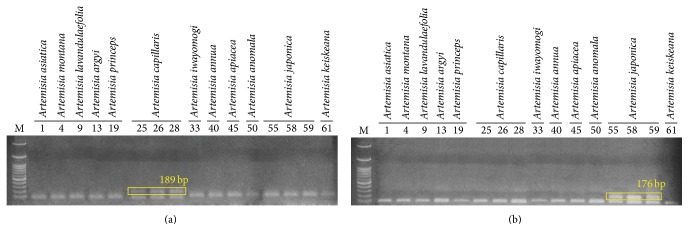
Polymerase chain reaction products of the primer sets AC F4/ACJ R3 (a) and AJ F1/AC R3 (b) from 12* Artemisia* species. Lane numbers are listed in Table 1. M: 100 bp ladder.

Based on the results shown in Figure 2, we could discriminate* A. capillaris* from other* Artemisia* species, at least from the 11* Artemisia* species used in this study, by differences in the ITS sequences. It was difficult to discriminate* A. capillaris* from* A. japonica*, which was close to* A. capillaris* on the dendrogram and exhibited significant variation in its ITS sequence. Most of the variation in the ITS sequences among the intraspecific* A. japonica* samples was found in the ITS2 region (Figure 1); therefore, we excluded the ITS2 region when designing primers to amplify specific DNA marker*s* for* A. japonica*. As shown in Figures 1 and 3, we designed the primer set AC F4/ACJ R3 in order to amplify a 189 bp PCR product in the ITS1 region that only appeared in* A. capillaris* samples (Figures 3 and 4(a)). Subsequently, we designed the AJ F1/AC R3 primer set in order to amplify a 176 bp PCR product in ITS2 that only appeared in* A. japonica* samples (Figures 3 and 4(b)).

Based on these results, we suggest that two primer sets (AJ F1/AC R3 and AC F4/ACJ R3) could be used to discriminate* A. capillaris* not only from* A. japonica* but also from other* Artemisia* species.

### 3.3. Discrimination of* Artemisia* Species That Are Sources of Artemisiae Annuae Herba and Artemisiae Anomalae Herba by Differences in ITS Sequences

**Figure 5:**
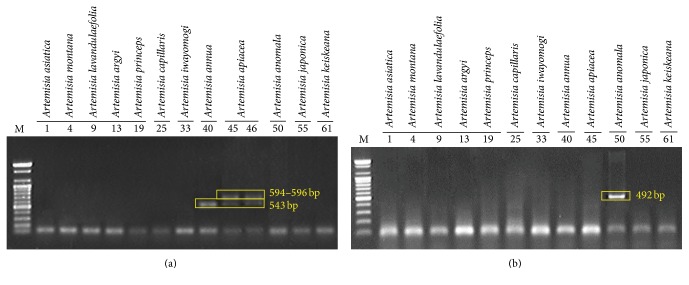
Polymerase chain reaction products of the primer sets AA F3/Aa R4 and Aap F1/Aap R2 (a) and Aam F3/Aa R4 (b) from 12* Artemisia* species. Lane numbers are listed in Table 1. M: 100 bp ladder.

We developed DNA markers in order to detect contamination of* A. capillaris* by other* Artemisia* species. As shown in Figure 2,* A. annua* and* A. apiacea*, which are sources of Artemisiae Annuae Herba, were close together on the dendrogram in a similar manner as* A. capillaris* and* A. japonica*. Therefore, we attempted to find region(s) in ITS1 and ITS2 to discriminate both* A. annua* and* A. apiacea* from other* Artemisia* species. As shown in Figures 1 and 3, we designed the AA F3/Aa R4 primer set in order to amplify a 543 bp PCR product in both* A. annua* and* A. apiacea* simultaneously as a common DNA marker. Subsequently, we designed primers to amplify a specific DNA marker to discriminate* A. annua* from* A. apiacea*. Based on the differences found in the ITS1 and ITS2 sequences, we designed the Aap F1/Aap R2 primer set in order to amplify a 594 (in sample number 45, which had a 2 bp deletion) or 596 bp (in sample number 46) PCR product that only appeared in* A. apiacea* samples (Figures 1 and 3). Based on amplifications of the one or two PCR products expected on the gel (Figure 5(a)), we confirmed that the AA F3/Aa R4 and Aap F1/Aap R2 primer sets could discriminate not only* A. annua* from* A. apiacea* but also these two species from other* Artemisia* species. In the case of* A. anomala*, we designed an Aam F3/Aa R4 primer set in order to amplify a 492 bp PCR product in* A. anomala* samples (Figures 1 and 3) and confirmed that the expected 492 bp single band of the PCR product only appeared in* A. anomala* samples (Figure 5(b)).

### 3.4. Detection of Contamination by Other* Artemisia* Species Using Multiplex PCR

**Figure 6:**
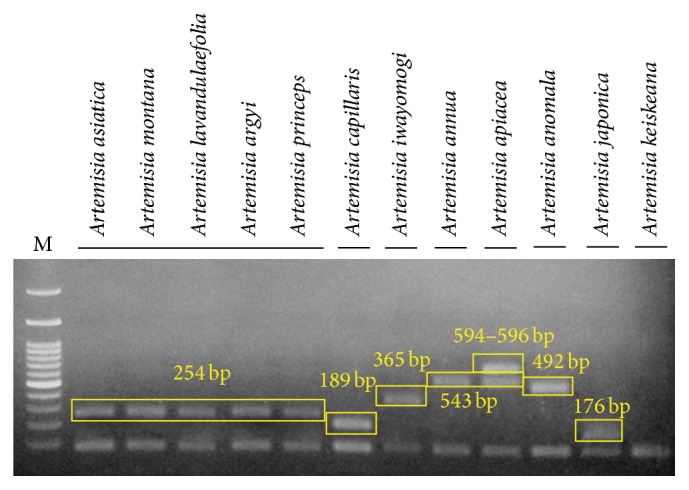
Multiplex polymerase chain reaction products using the primers shown in Figures 1 and 3 from 12 randomly selected* Artemisia* species. Genomic DNA from a randomly chosen sample of each* Artemisia* species was used for PCR amplification. M: 100 bp ladder.

As shown in Figures 1 and 2, differences in the ITS sequences could discriminate five* Artemisia* species—*A. asiatica*,* A. montana*,* A. lavandulaefolia*,* A. argyi*, and* A. princeps*—that are sources of Artemisiae Argyi Folium and* A. iwayomogi* that is a source of Artemisiae Iwayomogii Herba from the six other* Artemisia* species. However, designing primers in order to amplify DNA markers for these species based on differences in the ITS sequences was difficult. Therefore, we tested the usability of the Fb/R7 and 2F1/2F3 primer sets in order to amplify SCAR markers that were developed in previous studies with six* Artemisia* species [5, 7]. We confirmed that the Fb/R7 primer set amplified a 254 bp SCAR marker in samples of not only* A. princeps* and* A. argyi* but also* A. asiatica*,* A. lavandulaefolia*, and* A. montana* (data not shown). Furthermore, we confirmed that the 2F1/2F3 primer set amplified a 364 or 365 bp SCAR marker only in* A. iwayomogi*, and that this marker was not amplified in any other species, including* A. asiatica*,* A. montana*,* A. lavandulaefolia*,* A. annua*,* A. apiacea*, or* A. anomala* (data not shown). Therefore, these two RAPD-based primer sets could detect contamination by these* Artemisia* species in addition to the six other* Artemisia* species. Using the multiplex PCR method, we tested the reliability of these two primer sets and those developed based on the ITS sequences to discriminate* A. capillaris* from other* Artemisia* species and to detect contamination by other* Artemisia* species. For the multiplex PCR process, we randomly selected one sample from each* Artemisia* species listed in Table 1. As shown in Figure 6, these primer sets functioned reliably, not only to discriminate* A. capillaris* from other* Artemisia* species, but also to simultaneously detect contamination by other* Artemisia* species in a single PCR process.

**Figure 7:**
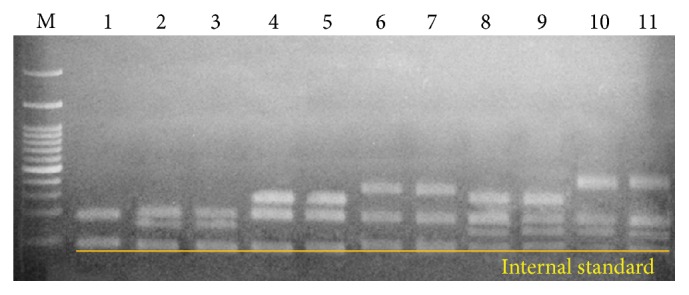
Multiplex polymerase chain reaction products by using two or three combined primer sets from mixed genomic DNA isolated from two or three* Artemisia* species at different content ratios. Primer set AC F4/ACJ R3 amplified a 189 bp DNA marker to detect* A. capillaris*; AJ F1/AC R3 amplified a 176 bp DNA marker to detect* A. japonica*; Fb/R7 amplified a 254 bp DNA marker to detect* A. princeps*; 2F1/2F3 amplified a 365 bp DNA marker to detect* A. iwayomogi*. Lane 1:* A. capillaris*; Lane 2:* A. capillaris* and* A. japonica* (9 : 1); Lane 3:* A. capillaris* and* A. japonica* (19 : 1); Lane 4:* A. capillaris* and* A. princeps* (9 : 1); Lane 5:* A. capillaris* and* A. princeps* (19 : 1); Lane 6:* A. capillaris* and* A. iwayomogi* (9 : 1); Lane 7:* A. capillaris* and* A. iwayomogi* (19 : 1); Lane 8:* A. capillaris*,* A. japonica*, and* A. princes* (8 : 1 : 1); Lane 9:* A. capillaris*,* A. japonica*, and* A. princeps* (18 : 1 : 1); Lane 10:* A. capillaris*,* A. japonica*, and* A. iwayomogi* (8 : 1 : 1); Lane 11:* A. capillaris*,* A. japonica*, and* A. iwayomogi* (18 : 1 : 1); M: 100 bp ladder, yellow underline: 96 bp internal standard amplified by AYF/AYR primer set.

Finally, by mixing genomic DNA isolated from different* Artemisia* species at varying content ratios, we tested the reliability of this PCR method to detect contamination of other* Artemisia* species, such as* A. japonica*,* A. princeps*, and* A. iwayomogi*, which are mostly found in Korea and are easily misused. As shown in Figure 7, the multiplex PCR detected two* Artemisia* species that had been mixed at ratios of 9 : 1 and 19 : 1. Furthermore, the multiplex PCR detected a mixture of three* Artemisia* species (*A. capillaris* with* A. japonica* and* A. princeps* or* A. capillaris* with* A. japonica* and* A. iwayomogi*) at ratios of 8 : 1 : 1 and 18 : 1 : 1 (Figure 7). Therefore, we suggest that the multiplex PCR method is an accurate tool to discriminate* A. capillaris* from other* Artemisia* species and could be used to determine whether* A. capillaris* samples have been mixed with other* Artemisia* species.

## 4. Discussion

Medicinal plants have long been used to treat disease in traditional and modern medicine [1]. However, because of the substitution and adulteration of medicinal plants with closely related species, the value of the original drug decreases and in some cases can make it lethal when substituted or contaminated with toxic adulterant plant(s) [3]. Therefore, the authentication of medicinal plants is crucial. As mentioned previously,* A. capillaris*, which is a source of Artemisiae Capillaris Herba, should be discriminated from not only* A. japonica*,* A. argyi*, and* A. princeps* but also other* Artemisia* species that grow and/or are cultivated in Korea and China and could contaminate the products of* A. capillaris*.* Artemisia* species, including* A. capillaris*, are a valuable source of new drugs and essential oils, and their unique chemical compositions and pharmacological activity are species-specific [1, 2, 5]. In this context, we developed a method to discriminate* A. capillaris* from other* Artemisia* species and to detect contamination among* Artemisia* species.

The DNA barcode is a powerful tool for identifying and discriminating between species of animal, plant, and fungus. The sequence at the 5′ end of cytochrome c oxidase subunit 1 (*CO1*) in the mitochondrial genome is used for animal taxonomic classification [25, 26]; however, plants cannot currently be identified by the sequence of a single locus [3]. Therefore, the Consortium for the Barcode of Life (CBOL) Plant Working Group proposed a combination of sequences of* matK* in the nuclear genome and* rbcL* in the chloroplast genome to identify plants [3]. However, the discriminatory power of the combined* matK* and* rblL* loci is low, particularly when discriminating between closely related species, such as 36 species in the genus* Dendrobium* [27]. Instead, by using a single* matK* sequence, medicinal plants in the subfamily Rauvolfioideae and genus* Rheum* have been successfully discriminated from each other [28, 29]. Despite the relatively low level of variation found in* rbcL* sequences in 48 plant genera including* Amaranthus*,* Angelica*, and* Ilex*, their combination with* trnH-psbA* intergenic spacer sequences increased the identification and discrimination success rate from 79% to 88% [30]. Therefore, to identify or discriminate between specific medicinal plants and closely related species, other single loci, besides* CO1*, or a combination of loci, besides* matK* and* rbcL*, have been used. For example, various* Dendrobium* Sw. species have been discriminated between them using a single sequence of the* trnH-psbA* intergenic spacer [31]. In addition, the* trnL-trnF* intergenic spacer sequence clearly discriminated* Cardiocrinum giganteum* from* C. giganteum* var. yunnanense and* C. cordatum* [32].

Of the various DNA barcode loci used, the ITS is one of the most useful. Multiple copies of the ITS are tandemly located at one or more chromosomal loci, and there are hundreds or thousands of ITS repeats in the nuclear genome. Furthermore, the ITS, including ITS1, 5.8S rRNA, and ITS2, is relatively small and ranges from 400 bp to under 1000 bp long [33]. Because of the presence of high copy numbers of the ITS and its small size, the ITS is easily amplified by PCR [34]. The level of variation among interspecific ITS sequences is high, so they can be used for the identification of plants at the specific, generic, and even family levels [35]. In contrast, levels of variation within intraspecific ITS sequences are often very low [34]. Concerted evolution should homogenize the sequences of ITS repeats that exist in a species by high-frequency unequal crossing over or gene conversion [36, 37]. The ITS2 sequence in particular has been used to identify medicinal plants that belong to the genera* Swartzia* and* Artemisia* in the family Fabaceae [13, 14]. In addition, ITS2 sequences, combined with* rbcL* sequences, have been used for detecting the contamination and substitution of products from 42 medicinal plants, including* Achillea racemose* and* Urtica dioica*, in Canada and the USA [38].

As shown in Figures 1 and 2, discriminating* A. capillaris* from* A. japonica* and 10 other* Artemisia* species was based upon differences in ITS sequences among the* Artemisia* species. Using the RAPD method with nonspecific primers, we were unable to discriminate* A. capillaris* from* A. japonica* in a previous study [5, 7]. Here, we were able to discriminate* A. capillaris* from* A. japonica* because of differences in nucleotide sequences, particularly in the ITS2 region (Figure 1). Basing discrimination on differences in ITS sequences was conducted cautiously, because of the considerable sequence variation found in the ITS sequences, particularly among intraspecific* A. japonica* samples. We also observed this variation in the* A. japonica* ITS sequences deposited in GenBank (accession numbers AM398882, AY548200, GU724289, JF326554, JX051713, and KC493078). Therefore, we confirmed the discriminatory power of the ITS sequences by using the* A. capillaris* and* A. japonica* ITS sequences deposited in GenBank. The deposited* A. japonica* sequences, together with the* A. japonica* sequences determined in this study (sample numbers 55–60), were clearly discriminated from both the deposited (accession numbers AY548201 and KC493083) and determined (sample numbers 25–32)* A. capillaris* sequences (data not shown).

Therefore, differences in ITS sequences can be used to discriminate among* Artemisia* species, despite the large variations observed in the ITS sequences of specific* Artemisia* species. Lee et al. [39] compared ITS sequences among* Artemisia* species that grow naturally in Korea, including two varieties and one subspecies of* A. japonica*. They estimated the pairwise divergence value as 0.004 between the varieties and subspecies based on the Kiura-2 parameter. Because we could not find any sequence information in their article or GenBank, we were unable to determine how many nucleotide variations exist between the varieties and subspecies of* A. japonica*. However, based on the results of their study, we suggest that the intraspecific ITS sequence variation detected in* A. japonica* could result from the different varieties and/or subspecies of* A. japonica* used for the determination of the ITS sequences.

For the discrimination of* A. capillaris* from* A. japonica*, which were closer to each other than to any other* Artemisia* species on the dendrogram (Figure 2), the primer sets AC F4/ACJ R3 (that amplified a 189 bp DNA marker in* A. capillaris*) and AJ F1/AC R3 (that amplified a 176 bp DNA marker in* A. japonica*) were designed (Figures 1 and 3). Despite the fact that there was not a remarkable difference in the sizes of the DNA markers for* A. capillaris* and* A. japonica*, they were clearly separated on 2% agarose gel after 40 min of gel running (Figure 6).

The method of amplifying double DNA markers of specific species was used to discriminate* A. annua* from* A. apiacea*, which were close to each other on the dendrogram (Figure 2). A 543 bp DNA marker was only amplified in* A. annua* using the AA F3/Aa R4 primer set, and both the 543 and 594 bp (or 596 bp, depending on the presence of a base deletion) DNA markers were amplified in* A. apiacea* using the AA F3/Aa R4 and Aap F1/Aap R2 primer sets, respectively (Figures 5(a) and 5(b)). For the discrimination of the five* Artemisia* species that are sources of Artemisiae Argyi Folium, we first determined whether the nonspecific UBC primer 329 (5′-GCGAACCTCC-3′), which amplified a unique 850 bp PCR product only in* A. argyi* and* A. princeps* in a previous study [7], could amplify the same PCR product in three additional* Artemisia* species (*A. asiatica*,* A. montana*, and* A. lavandulaefolia*). Using samples from the 12 species, we confirmed that the same PCR products were amplified in these three* Artemisia* species (data not shown). We then confirmed that the Fb/R7 primer set, which was designed to amplify a 254 bp SCAR marker based on the sequence of an 850 bp PCR product [7], amplified the same-sized DNA marker in the three additional* Artemisia* species (data not shown). For the discrimination of* A. iwayomogi*, we tested whether the nonspecific UBC primer 391 (5′-GCGAACCTCG-3′), which amplified four kinds of PCR products that ranged in size from 707 to 719 bp in* A. iwayomogi* in a previous study [5], could amplify the same PCR products in six additional* Artemisia* species (*A. asiatica*,* A. montana*,* A. lavandulaefolia*,* A. apiacea*,* A. annua*, and* A. anomala*). We confirmed that the UBC primer 391 amplified PCR products only in* A. iwayomogi*. In addition, we confirmed that the 2F1/2F3 primer set, which was designed to amplify a 365 bp SCAR marker based on the sequences of four PCR products [5], amplified the same-sized DNA marker only in* A. iwayomogi* (data not shown). Based on these results, we were convinced that the Fb/R7 and 2F1/2F3 primer sets could discriminate* A. capillaris* not only from the five* Artemisia* species that are sources of Artemisiae Argyi Folium but also from* A. iwayomogi*.

Using primer sets based on the ITS sequences and RAPD results to discriminate among the* Artemisia* species, we evaluated the multiplex PCR method to discriminate* A. capillaris* and to detect contamination of* A. capillaris* by randomly selecting each sample of* Artemisia* species (Figure 6) and mixed samples of* A. capillaris* with* A. japonica*,* A. princeps*, and* A. iwayomogi* (Figure 7). Therefore, we suggest that the multiplex PCR method is an accurate tool to discriminate* A. capillaris* from other* Artemisia* species and could be used to determine whether* A. capillaris* samples have been mixed with samples from other* Artemisia* species, at least those tested in this study.

## 5. Conclusion

To differentiate among* A. capillaris* plants that produce Artemisiae Capillaris Herba and 11 other* Artemisia* species, 726–731 bp ITS nucleotide sequences in 65 samples were determined and analyzed. Based on differences found in partial ITS nucleotide sequences between the species, we designed the primer sets AC F4/ACJ R3 to amplify a 189 bp PCR product and AJ F1/AC R3 to amplify a 176 bp PCR product in* A. capillaris* and* A. japonica*, respectively. To detect traces of other* Artemisia* species in* A. capillaris*, we designed the primer set AA F3/Aa R4 to amplify a 543 bp product in* A. annua*, the primer set Aap F1/Aap R2 to amplify a 594–596 bp product in* A. apiacea*, and the primer set Aam F3/Aa R4 to amplify a 492 bp product in* A. anomala*. In addition, we confirmed that the primer sets Fb/R7 and 2F1/2F3, which had been developed in a previous study based on RAPD, could be used to amplify 254 bp products in* A. princeps*,* A. argyi*,* A. asiatica*,* A. lavandulaefolia*, and* A. montana*, which are sources of Artemisiae Argyi Folium, and to amplify 364 or 365 bp products in* A. iwayomogi*. Therefore, we demonstrate that the discrimination of* A. capillaris* from and the detection of contamination by other* Artemisia* species can be reliably performed by multiplex PCR using these primers.

## Figures and Tables

**Table 1:** * Artemisia* plants used to determine the internal transcribed spacer (ITS) sequence.

Number	Medicinal name	Name of the plant species	Place of collection	Voucher number

1	Artemisiae Argyi Folium	*A. asiatica*	Bonghwa, Korea	WKUARE04
2				WKUARE24
3				WKUARE25
4		*A. montana*	Kyoto, Japan	WKUARE76
5				WKUARE77
6				WKUARE78
7			Jeonju, Korea	WKUARE40
8				WKUARE41
9		*A. lavandulaefolia*	Sichuan, China	WKUARE66
10				WKUARE57
11				WKUARE58
12				WKUARE67
13		*A. argyi*	Suwon, Korea	WKUARE05
14				WKUARE06
15				WKUARE30
16				WKUARE31
17			Guangxi, China	WKUARE27
18			Sichuan, China	WKUARE59
19		*A. princeps*	Jeonju, Korea	WKUARE43
20				WKUARE44
21				WKUARE45
22			Uiseong, Korea	WKUARE01
23			Ganghwa, Korea	WKUARE55
24				WKUARE56

25	Artemisiae Capillaris Herba	*A. capillaris*	Suwon, Korea	WKUARE33
26				WKUARE34
27			Jeonju, Korea	WKUARE35
28				WKUARE46
29				WKUARE47
30			Nishi, Japan	WKUARE79
31				WKUARE80
32			Sichuan, China	WKUARE52

33	Artemisiae Iwayomogii Herba	*A. iwayomogi*	Suwon, Korea	WKUARE37
34			Jinan, Korea	WKUARE10
35			Pohang, Korea	WKUARE68
36				WKUARE69
37			Jeonju, Korea	WKUARE11
38				WKUARE48
39				WKUARE49

40	Artemisiae Annuae Herba	*A. annua*	Namwon, Korea	WKUARE20
41			Yeongcheon, Korea	WKUARE21
42			Sichuan, China	WKUARE60
43				WKUARE61
44				WKUARE62
45		*A. apiacea*	Sichuan, China	WKUARE63
46				WKUARE53
47				WKUARE54
48			Nishi, Japan	WKUARE81
49				WKUARE82

50	Artemisiae Anomalae Herba	*A. anomala*	Sichuan, China	WKUARE64
51				WKUARE65
52			Pohang, Korea	WKUARE73
53				WKUARE74
54				WKUARE75

55	Artemisiae Japonicae Herba	*A. japonica*	Suwon, Korea	WKUARE39
56			Jeonju, Korea	WKUARE50
57				WKUARE51
58			Namryung, China	WKUARE17
59			Nishi, Japan	WKUARE83
60				WKUARE84

61	Artemisia Keiskeanae Herba	*A. keiskeana*	Suwon, Korea	WKUARE16
62			Uiseong, Korea	WKUARE15
63			Pohang, Korea	WKUARE70
64				WKUARE71
65				WKUARE72
